# Pollution Haven Hypothesis of Global CO_2_, SO_2_, NO_x_—Evidence from 43 Economies and 56 Sectors

**DOI:** 10.3390/ijerph18126552

**Published:** 2021-06-18

**Authors:** Ke Zhang, Xingwei Wang

**Affiliations:** College of Control Science and Engineering, China University of Petroleum (East China), Qingdao 266580, China; ZhangKe_0404@163.com

**Keywords:** pollution haven hypothesis, multi-regional input-output model, CO_2_ (carbon dioxide), SO_2_ (sulfur dioxide), NO_x_ (nitrogen oxides)

## Abstract

With the development of trade liberalization, the pollutants emissions embodied in global trade are increasing. The pollution haven hypothesis caused by trade has aroused wide attention. The fragmentation of international production has reshaped trade patterns. The proportion of intermediate product trade in global trade is increasing. However, little has been done to study the pollution haven of different pollutants under different trade patterns. In this paper, major environmental pollutants CO_2_ (carbon dioxide), SO_2_ (sulfur dioxide), and NO_x_ (nitrogen oxides) are selected as the research objects. This study investigated the global pollution haven phenomenon in 43 countries and 56 major industries from 2000 to 2014. Based on the MRIO model, the trade mode is divided into three specific patterns: final product trade, intermediate product trade in the last stage of production, and the trade related to the global value chain. The results show that trade liberalization could reduce global CO_2_, SO_2_, and NOx emissions, and intermediate product trade has a more significant emission reduction effect than final product trade. Trade’s impacts on each country are various, and the main drivers are also different. For example, the European Union avoids becoming a pollution haven mainly through the trade related to the global value chain. The suppressed emissions under this trade pattern are 71.8 Mt CO_2_, 2.2 Mt SO_2_, 2.2 Mt NO_x_. India avoids most pollutants emissions through intermediate product trade. China has become the most serious pollution haven through final product trade. The trade pattern could increase China 829.4 Mt CO_2_, 4.5 Mt SO_2_, 2.6 Mt NO_x_ emissions in 2014.

## 1. Introduction

In the past few decades, global trade has grown rapidly [1], and trade has an increasing impact on resource flows and pollutant transfers among countries [2]. The pollutants embodied in international trade are also increasing [3], which has raised people’s concerns about the environmental problems caused by trade.

There have been some debates among scholars about the environmental consequences of trade liberalization [4]. The pollution haven hypothesis is the most significant one [5]. In order to promote economic growth, some countries may lower environmental standards to attract pollution-intensive industries, which will lead to the transfer of pollutants emissions [6]. If this hypothesis is true, high-standard countries’ environment may be improved, but from a global perspective, the world’s total pollutants emissions may increase. Therefore, through the assessment of the pollution haven phenomenon, we could understand the emission responsibility of each country and promote global trade’s coordinated and sustainable development.

Most existing studies have analyzed the pollution haven of CO_2_ [1,7,8,9]. SO_2_ and NO_x_ are also major environmental pollutants in the atmosphere, but little has been done about them. This paper studies the pollution haven of CO_2_, SO_2_, and NOx. With the deepening of production fragmentation, intermediate product trade has played an increasingly important role in global trade [10], and the pollution haven hypothesis has become more complex [1]. In order to reveal the impact of international production fragmentation on the environment, this paper divides trade into three patterns: final product trade, intermediate product trade in the last stage of production, and the trade related to the global value chain, so as to study the pollution haven phenomenon under the three patterns.

Based on the environmentally extended MRIO model, this paper assesses the pollution haven phenomenon of global CO_2_, SO_2_, and NOx emissions in three specific trade patterns. There are three main findings: (1) overall, the trade could reduce global emissions of CO_2_, SO_2_, and NOx; (2) intermediate product trade shows a more significant reduction effect than final product trade during the period 2000–2014; and (3) most countries avoid becoming a pollution haven through trade, but the main drivers are different from countries. For example, the European Union avoids becoming a pollution haven mainly through the trade related to the global value chain. China becomes the worst pollution haven through final product trade.

The remainder of this paper is structured as: Section 2 reviews the related studies about pollutions haven, presents the innovation of the study, Section 3 explains the modeling process and data sources, Section 4 presents the main results and discussions, and Section 5 summarizes the main findings and limitations of the study.

## 2. Literature Review

In recent years, a large number of air pollutants CO_2_, SO_2_, and NOx have been reallocated, posing an increasing threat to the ecological environment and human health [11,12,13,14]. The rapid development of trade globalization has promoted the global flows of pollutants, which has aroused people’s concern about the plight of trade-induced environmental problems. The share of pollutants emitted by international trade has been increasing over time [2]. Zhang et al. found that in 2009, about 25% of global carbon emissions were caused by international trade [5]. The emissions embodied in trade cover the goods’ entire production process: from raw materials acquisition, manufacturing, processing, and transportation to the final product in the hands of consumers [15]. With the development of economic globalization, countries’ active participation in the global labor division will lead to the transfer of pollutants emissions [16]. The pollution haven hypothesis is an important issue on the environmental problems caused by international trade. Pollution haven means that international trade could increase global pollutants emissions [17]. Developing countries tend to attract developed countries’ investment by virtue of their low labor and low environmental costs. In order to pursue the maximization of profits, developed countries often spend less on investment and transfer their pollution-intensive enterprises to developing countries with lower prices. These developing countries assume the pollutants emissions responsibility and become pollution haven in global trade [18]. To make matters worse, if the pollution-intensive industries’ efficiency in these developing countries is low, the world’s total emissions will increase due to trade diversion [19]. Scholars have worked on various aspects of the pollution haven hypothesis over the years: Duan et al. studied the role of multinational enterprises in global carbon emissions [20] Himics et al. researched trade liberalization’s effect on CO_2_ emissions in agriculture [21,22]. There are also some arguments about the pollution haven: Che et al. found that China became a pollution haven in international trade [23]. However, Zhang et al. found that China’s exports are becoming cleaner due to globalized production [24]. Xu et al. pointed out that trade liberalization reduces smog emissions [25]. Therefore, it is of great significance to assess the pollution haven hypothesis. However, at present, most scholars have studied the pollution haven phenomenon of CO_2_ from various perspectives [7,8,9], and few scholars have taken both SO_2_ and NO_x_ into consideration. Therefore, this study takes major environmental pollutants CO_2_, SO_2_, and NOx as research objects to assess pollution haven.

Studies have found that the multi-regional input-output model could describe the close correlation among countries and sectors [26], and a large number of scholars have applied it in the environmental issues related to the global value chain. Lu et al. calculated the evolution trend of China’s SO_2_ emissions from the consumption side [11]. Lu et al. found some pollution-intensive sectors in China have been phased out to the Belt and Road Routes countries [27]. Jiang et al. studied the U.S.’s oil footprint drivers [28]. Chen et al. found global trade exacerbates land and virtual water’s uneven distribution [29]. Chen et al. analyzed global energy flows by using the environmental-extend input-output model [30]. In addition, compared with the SRIO model, the MRIO model could reduce the measurement error [31]. In the MRIO model, the imported products and domestic products come from different places [32]. It could be seen that the MRIO model has great advantages in the research and is widely used. Therefore, this study uses the MRIO model to study CO_2_, SO_2_, and NOx emissions embodied in global trade from 2000 to 2014.

For the past few years, economic globalization’s most prominent feature is production fragmentation in the global value chain [32]. Fragmentation means that the production process is broken down into different stages, and each country on the value chain produces a specific part. These countries are linked to each other through trade. The fragmentation could reshape trade patterns and optimize international labor division but redistribute trade-related emissions at the same time [33]. For example, it is found that there is a shift in recent years’ global supply chain, and it means the transfer of resources and pollution [34]. Yilin et al. found carbon emissions were redistributed in the current complex global trade network [35]. In previous studies, Wang et al. studied the carbon emissions in China–Australia trade [36]. Araújo et al. found that Brazil played the role of the intermediate supplier in global value chains [37]. Scholars usually focused on bilateral trade or the role of one country in international production decentralization, but few assessed the composition of global value chains and the pollution haven of different trade patterns. Therefore, this paper studies the pollution haven hypothesis from the perspective of global state composition so that each country’s contribution to the global pollution haven could be clearly seen in the results, which are more in line with the needs of policymakers. In addition, Zhang et al. distinguished three specific trade patterns in the international trade production division: final product trade, intermediate product trade in the last stage of production, and the trade related to the global value chain, and they studied the carbon pollution haven in three trade patterns [5]. Wang et al. also studied China’s environmental impact of PM_2.5_ in different trade patterns [32]. On the basis of previous studies, this study further refines research contents to the industry level and studies emissions responsibility and pollution haven of different industries and patterns.

## 3. Method and Data

### 3.1. Multi-Regional Input-Output Analysis

The input-output method was proposed by Leontief in the 1930s [38]. It has been widely used to analyze the economic activities so as to reveal the internal relationship among industries of the national economy and do some economic forecasts and arrangements.

Table 1 is the basic form of the multi-regional input-output model (MRIO). There are m countries (regions) in the multi-regional input-output model, and each country has n sectors. The rows in the table represent the distribution of intermediate product trade and final demand in each country’s industry, and the columns represent intermediate input from different countries and industries.

The first quadrant, *Z*, reflects the direct economic linkages between the various industries of countries. $Z_{ij}^{pq}$ denotes the intermediate input from country *p*’s sector *i* to country *q*’s sector *j*, which needs to be reprocessed before they can be consumed [39]. That is the intermediate demand matrix in the input-output table provided by the WIOD database: a 2464*2464 column vector formed by 44 regions and 56 sectors.

The second quadrant *Y* reflects the final demand matrix. $Y_{j}^{pq}$ denotes country *q*’s final demand for goods and services from country *p*’s sector *j*, which could be consumed directly [40]. It is the final demand matrix in the input-output table: a 2464*1 column vector consisting of 44 regions, 56 sectors, and 5 final requirements.

The third quadrant *V =* $\left[V_{j}^{q}\right]$ reflects the added value of each country [41]. The horizontal summation $X^{q}=\left[X_{j}^{q}\right]$ is the total output matrix of country *q*. It is the last column in the input-output table: a 2464*1 column vector formed by 44 regions and 56 sectors.

$A^{pq}$ is the direct coefficient consumption matrix that represents the direct consumption of goods or services for unit total output in various sectors [42], $A^{pq}=\left[A_{ij}^{pq}\right]$, $A_{ij}^{pq}=\frac{Z_{ij}^{pq}}{X_{j}^{q}}$. Then, the total output of a country can be expressed as:

(1) X=AX+Y

(2) $$\left[\begin{array}{c} X^{1}\\ X^{2}\\ \vdots \\ X^{m} \end{array}\right]=\left[\begin{array}{c} A^{11}\;\;A^{12}\;\;\ldots \;A^{1m}\;\\ A^{21}\;\;A^{22}\;\;\ldots \;A^{2m}\\ \vdots \;\vdots \;\ddots \;\vdots \\ A^{m1}\;\;A^{m2}\;\;\ldots \;A^{mm} \end{array}\right]\left[\begin{array}{c} X^{1}\\ X^{2}\\ \vdots \\ X^{m} \end{array}\right]+\left[\begin{array}{c} \sum_{q=1}^{M}Y^{1q}\\ \sum_{q=1}^{M}Y^{2q}\\ \vdots \\ \sum_{q=1}^{M}Y^{mq} \end{array}\right]$$

A variant equation is as follows:

(3) $$\left[\begin{array}{c} X^{1}\\ X^{2}\\ \vdots \\ X^{m} \end{array}\right]={\left[\begin{array}{c} I-A^{11}\;-A^{12}\;\ldots \;-A^{1m}\;\\ -A^{21}\;I-A^{22}\;\ldots \;-A^{2m}\\ \vdots \;\vdots \;\ddots \;\vdots \\ -A^{m1}\;-A^{m2}\;\ldots \;I-A^{mm} \end{array}\right]}^{-1}\left[\begin{array}{c} \sum_{q=1}^{M}Y^{1q}\\ \sum_{q=1}^{M}Y^{2q}\\ \vdots \\ \sum_{q=1}^{M}Y^{mq} \end{array}\right]=\left[\begin{array}{c} B^{11}\;\;B^{12}\;\ldots \;B^{1m}\;\\ B^{21}\;\;B^{22}\;\ldots \;B^{2m}\\ \;\;\vdots \;\vdots \;\ddots \;\vdots \\ B^{m1}\;B^{m2}\;\ldots \;B^{mm} \end{array}\right]\left[\begin{array}{c} \sum_{q=1}^{M}Y^{1q}\\ \sum_{q=1}^{M}Y^{2q}\\ \vdots \\ \sum_{q=1}^{M}Y^{mq} \end{array}\right]$$

where $B^{pq}={\left(I-A^{pq}\right)}^{-1}$ is the Leontief inverse matrix [43]. It is also called the completed consumption coefficient matrix, meaning country *q*’s direct and indirect demand for country *p*’s commodities and services to produce the unit output.

The total exports from country *p* to country *q* could be expressed as:

(4) $$T^{pq}=Y^{pq}+Z^{pq}=Y^{pq}+A^{pq}X^{p}$$

According to Wang’s study [44], it could be proved that

(5) $$B^{qq}=L^{qq}+L^{qq}\sum_{s\neq q}^{M}A^{qs}B^{sq}$$

Combine (4) and (5) to obtain

(6) $$T^{pq}=Y^{pq}+A^{pq}X^{p}\;\;\;T^{pq}=Y^{pq}+A^{pq}\left[L^{qq}Y^{qq}+L^{qq}\underset{s\neq q}{\sum }A^{qs}B^{sq}Y^{qq}+\underset{s\neq q}{\sum }B^{qs}Y^{sq}+\underset{s}{\sum }B^{qs}\underset{r\neq q}{\sum }Y^{sr}\right]\;=\underset{T_{f}^{pq}}{\underbrace{Y^{pq}}}+\underset{T_{i}^{pq}}{\underbrace{A^{pq}L^{qq}Y^{qq}}}+\underset{T_{g}^{pq}}{\underbrace{A^{pq}L^{qq}\sum_{s\neq q}A^{qs}B^{sq}Y^{qq}+A^{pq}\sum_{s\neq q}B^{qs}Y^{sq}+A^{pq}\sum_{s}B^{qs}\sum_{r\neq q}Y^{sr}}}$$

$T_{f}^{pq}$ represents the final product trade exported from county *p* to country *q*.

$T_{i}^{pq}$ represents that country *p* provides intermediate product to country *q*, and country *q* reprocesses intermediate product itself to meet domestic final demand $Y^{qq}$, that is, the intermediate product trade in the last stage of production.

$T_{g}^{pq}$ represents the trade related to the global value chain [45], involving the remaining production process of semi-finished and finished products. The product crosses countries many times and may eventually be absorbed by final consumers [46].

Based on the above analysis, the total output of country *p* can be decomposed from the production side:

(7) $$X^{p}=A^{pp}X^{p}+Y^{pp}+\underset{p\neq q}{\sum }T^{pq}$$

A variant equation is as follows:

(8) $$\left(I-A^{pp}\right)X^{p}=Y^{pp}+\underset{p\neq q}{\sum }T^{pq}$$

(9) $$X^{p}=L^{pp}(Y^{pp}+\underset{p\neq q}{\sum }T^{pq})$$

(10) $$X^{p}=L^{pp}Y^{pp}+L^{pp}\underset{p\neq q}{\sum }T_{f}^{pq}+L^{pp}\underset{p\neq q}{\sum }T_{i}^{pq}+L^{pp}\underset{p\neq q}{\sum }T_{g}^{pq}$$

As can be seen from the above equation, the total output of country p is divided into four items: the first item represents domestic final demand, the second item represents direct export of final product trade, the third item is the direct intermediate product trade export, namely intermediate product trade export in the final stage, and the fourth item represents intermediate product trade export related to the value chain.

We define that pollutant emissions intensity coefficient of country *p* is represented by $f^{p}$ [47], $f^{p}=\frac{e^{p}}{x^{p}}$. It is the column vector of the environment variable in the environment account divide by the total output column vector in the input-output table. $e^{p}$ represents the pollutant emissions of country *p*. $x^{p}$ represents the gross output of country *p*.

(11) $$F^{p}=\left[f^{p}\right]$$

Based on the MRIO model and the above analysis, the total pollutant emission composition of country *p* can be expressed as:

(12) $$E^{p}=F^{p}X^{pp}=F^{p}L^{pp}Y^{pp}+\underset{F^{pp}L^{pp}T}{\underbrace{F^{p}L^{pp}\underset{p\neq q}{\sum }T_{f}^{pq}+F^{p}L^{pp}\underset{p\neq q}{\sum }T_{i}^{pq}+F^{p}L^{pp}\underset{p\neq q}{\sum }T_{g}^{pq}}}$$

**Figure 1:**
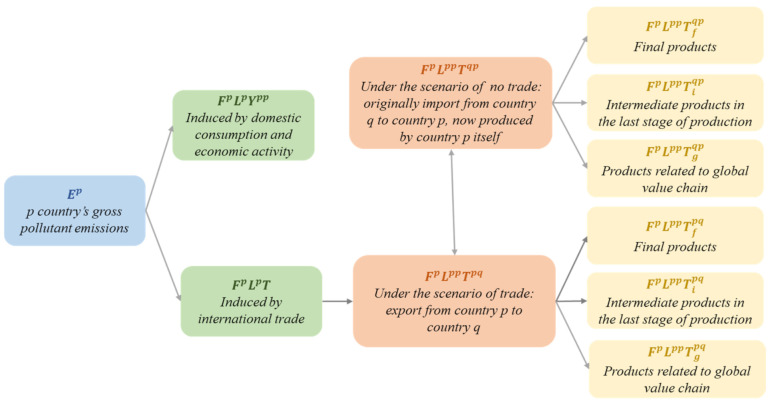
The decomposition of country p’s gross pollutant emissions.

$E^{p}$ can be divided into two parts: $F^{p}L^{pp}Y^{pp}$ is induced by domestic consumption and economic activities, and $F^{p}L^{pp}T$ is induced by international trade. The decomposition of country p’s gross pollutant emissions can be seen in Figure 1.

For the pollutant emissions induced by international trade $F^{p}L^{pp}T$:

Under the scenario of trade, pollutant emissions of country *p* are caused by exports from country p to other countries:

(13) $$EWT^{pq}=F^{p}L^{pp}T^{pq}=F^{p}L^{pp}T_{f}^{pq}+F^{p}L^{pp}T_{i}^{pq}+F^{p}L^{pp}T_{g}^{pq}$$

Under the scenario of no trade, pollutant emissions in country *p* induced by domestic production of commodities that are originally imported from other countries:

(14) $$ENT^{pq}=F^{p}L^{pp}T^{qp}=F^{p}L^{pp}T_{f}^{qp}+F^{p}L^{pp}T_{i}^{qp}+F^{p}L^{pp}T_{g}^{qp}\;\;$$

*ET* represents the total emissions difference between trade (*EWT*) and no-trade (*ENT*) scenarios:

*ET* = *EWT* − *ENT*

Similarly, according to the above derivation, *ET* can be decomposed as:

(15) $$ET=ET_{f}+ET_{i}+ET_{g}$$

$ET_{f}$ represents the final product trade emissions difference between trade and no-trade scenarios. $ET_{i}$ represents the emissions difference of intermediate product trade in the last stage of production. $ET_{g}$ represents the difference of the trade related to the global value chain.

### 3.2. Data Collection and Treatment

The data used in this paper includes two parts: input-output table and environmental pollutants emissions. The input-output table is derived from the latest world input-output database (WIOD 2016), which covers 43 countries (regions) and one rest of world (ROW), and 56 industries in each country [48]. Compared with the 2013 version, WIOD released in 2016 covers a wider range of countries and a more detailed industry classification [49]. WIOD database is also widely used in the field of input-output and environmental footprint tracking [50,51,52]. The newly released WIOD only provides environmental data of CO_2_, while the Eora database covers more than 2000 environmental indicators. Among these indicators, CO_2_, SO_2_, and NO_x_ are the main pollutants that cause environmental pollution problems, so the data of CO_2_, SO_2_, and NO_x_ in Eora is selected for research. In order to match the WIOD input-output table, this paper adjusted the Eora emission data to the WIOD form: 190 economies are merged into 44 economies, and 26 industries are disaggregated into 56 sectors according to the output structure. To better describe the results, the study combined the EU 28 countries into one economy EU-28 and combined 56 sectors into 7 main industries. The scope of this study is from 2000 to 2014, which covers some important events in the world: China’s accession to the WTO in 2001, the global economic crisis in 2008, and the shale gas revolution in the U.S. after 2009. In addition, in order to reduce the impact of inflation, WIOD data used in the study are levelized in 2010 constant dollar value according to the price index and exchange rate.

## 4. Discussion

### 4.1. Situation of the Pollutants Embodied in Trade

#### 4.1.1. Changing Trends of the Emissions under Three Trade Patterns

Overall, pollutants emissions caused by global trade increased from 2000 to 2014. In 2000, global emissions of CO_2_, SO_2_, and NO_x_ in trade were 5.4 billion tons, 45.2 million tons, and 43.0 million tons, while in 2014, they increased to 8.2 billion tons, 56.6 million tons, and 51.5 million tons. The changes mean that while international trade promotes economic development, it also increases air pollutants emissions and brings a huge environmental burden.

The emissions caused by intermediate product trade are much higher than final product trade, and intermediate product trade is more susceptible to the economic environment. In the research, intermediate product trade in the last stage of production and the trade related to the global value chain could be collectively called intermediate product trade [53]. In 2014, the emissions of CO_2_, SO_2_, and NO_x_ caused by the final product trade were about 2.9 billion tons, 18.9 million tons, and 17.7 million tons. The emissions of CO_2_, SO_2_, and NO_x_ caused by intermediate product trade are about 5.3 million tons, 37.7 million tons, and 33.8 million tons, which are nearly two times of final product trade. The global financial crisis in 2008 has an impact on the world economic and trade system, so emissions fell greatly in 2009. Final product trade’s CO_2_, SO_2_, and NO_x_ emissions decreased about 270 million tons, 1.3 million tons, 1.2 million tons, and intermediate product trade of each emission decreased about 710 million tons, 4.0 million tons, 3.5 million tons, which suggests that intermediate product trade is much more affected by the global economy than final product trade.

**Figure 2:**
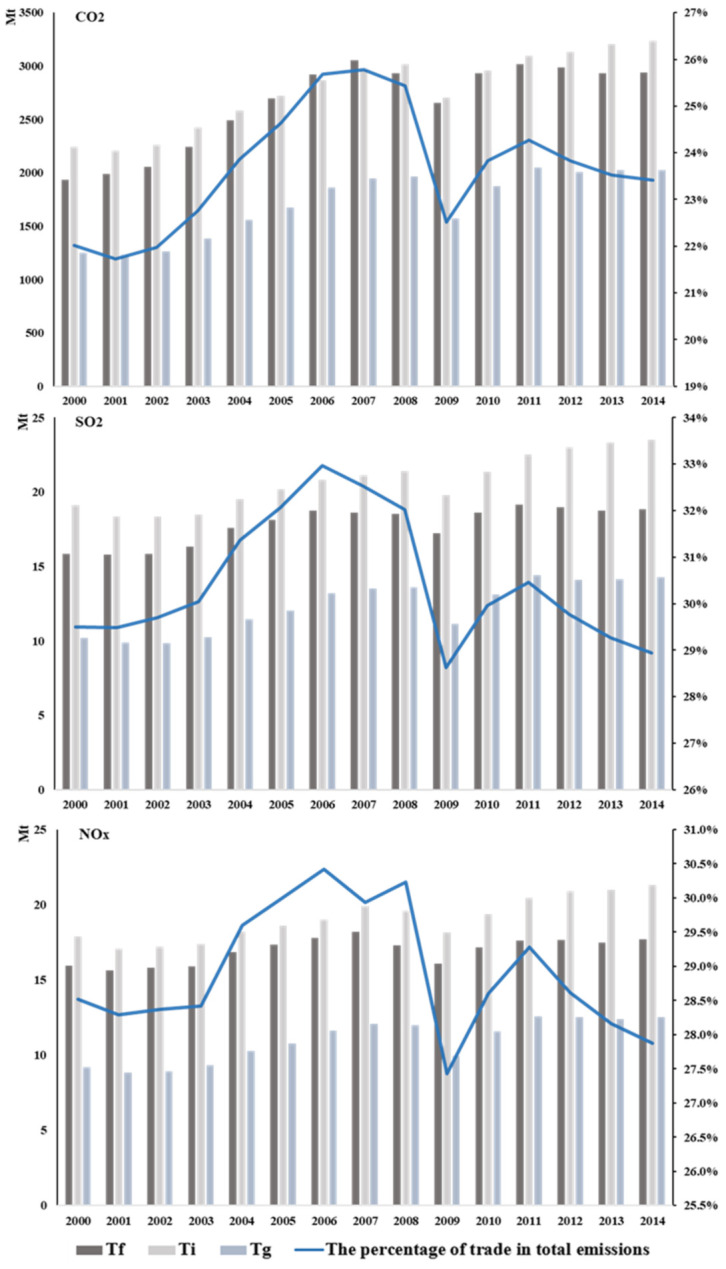
Changes in emissions under the three trade patterns in 2000–2014. The x-axis represents years; the left-hand-axis represents pollutants emissions under different trade patterns: Tf—final product trade; Ti—intermediate product trade in the last stage of production; Tg—the trade related to the global value chain. The right-hand axis represents the percentage of trade in total emissions. See Appendix C Table A3 for details.

CO_2_, SO_2_, and NO_x_ have similar trends because air pollutants and greenhouse gases have the same root homology. As can be seen from Figure 2, the proportion of emissions caused by trade increased gradually before 2007. However, due to the impact of the international financial crisis in 2008, the global trade was tightened, the proportion decreased. After that, the proportion raised again.

#### 4.1.2. Regional Situations of Pollutants Emissions

The global total emissions of CO_2_, SO_2_, and NO_x_ are about 35 billion, 190 million, and 180 million tons in 2014. China, the U.S., the European Union, and India are major pollutants sources. The emissions of CO_2_, SO_2_, and NO_x_ caused by international trade account for about a quarter of their total emissions. The final product trade pattern accounts for about 10%, intermediate product trade in the last stage of production is about 11%, trade of global value chain accounts for about 6%.

**Figure 3:**
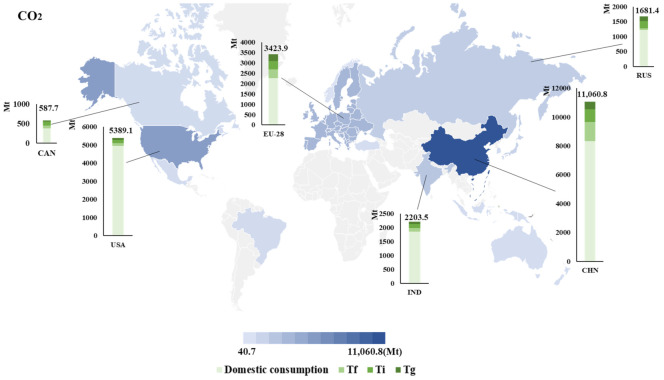

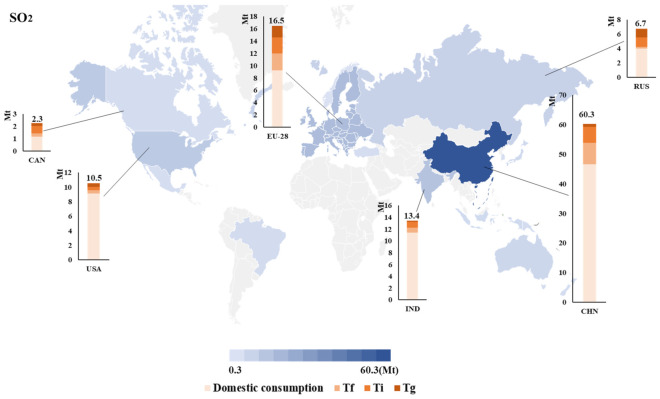

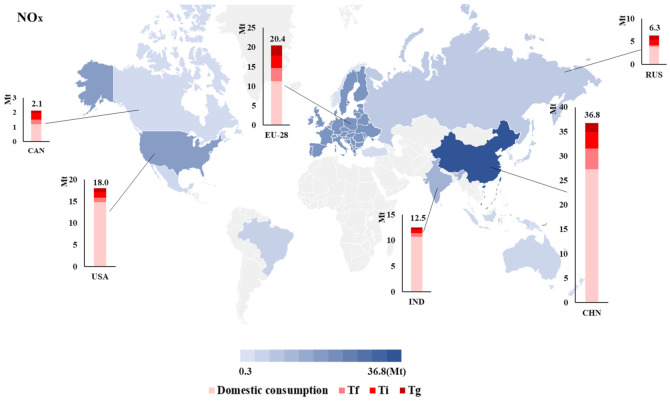
The situation of global pollutants emissions in 2014. Fifteen major regions are selected. See Appendix A Table A1, Table A2, Table A3 and Table A4 for detailed information.

As can be seen from Figure 3, China is the largest emitter of the three pollutants. Its emissions are far ahead of other countries, and the final product trade pattern in China produces the most pollutants. About 31.6% CO_2_, 31.5% SO_2_, and 20.0% NO_x_ of global emissions come from China. For China, about a quarter of its emission is induced by international trade. Specifically, final product trade produces the most pollutants. For example, in 2014, China’s CO_2_ emissions caused by the final product trade are 1.3 billion tons, far more than the emissions caused by intermediate product trade in the last stage of production and the trade related to the global value chain. It might because China takes on a lot of downstream production, processes intermediate goods, and then exports them to countries with final demand.

The proportion of pollutants emissions caused by international trade in the U.S. is lower than the world average, and most of them come from intermediate product trade. In 2014, the total emissions of CO_2_, SO_2_, and NO_x_ in the U.S. were 5.4 billion tons, 10.5 million tons, and 18.0 million tons. The emissions caused by international trade only account for 8.1%, 13.5%, and 17.5% of the total emissions, all lower than the world average level. However, for the U.S. itself, intermediate product trade causes more emissions. The emissions of CO_2_, SO_2_, and NO_x_ induced by this trade pattern are about 280 million tons, 1 million tons, and 2 million tons, which is about two times that of the final product trade. This may be because the U.S. is engaged in the high-value-added labor division in upstream production. The level of science and technology in the U.S. is advanced, and the country tends to export high-value-added intermediate products.

About 15% of India’s emissions come from international trade, mainly through traditional Ricardian trade patterns. The final product trade and the intermediate product trade in the last stage of production are called the traditional Ricardian trade pattern or direct-value-added trade pattern [45,54]. In 2014, India’s emissions of CO_2_, SO_2_, and NO_x_ were 2.2 billion tons, 13.4 million tons, and 12.5 million tons. Traditional Ricardian trade pattern accounts for about 12%, while the trade related to global value chain accounts for only about 3%. It might because India is a developing country with a low level of production. There is still a big gap compared with developed countries in this aspect, so the trade related to the global value chain takes up a small proportion.

The European Union’s emissions caused by international trade account for a much higher proportion than the world average, and the emissions caused by the three trade patterns are basically balanced. In 2014, the proportion of CO_2_, SO_2_, and NO_x_ caused by trade account for 33.7%, 43.7%, and 45.2% of the total emissions, while the world average level was about 25%, both higher than the world average level. This may be because the EU’s economy is developed and has a high level of technology. It usually takes advantage of these superiorities and actively participates in international trade, so its emissions caused by global trade are higher [55].

In Brazil, pollutants emissions caused by international trade are 66.7 Mt CO_2_, 0.4 Mt SO_2_, and 0.8 Mt NO_x_. The emissions caused by trade account for 12%, 16%, and 17% of the total emissions. The country’s share of emissions from international trade is below world levels. Among the three trade patterns, the intermediate product trade in the last stage of production produces the most emissions. About 42 million tons of CO_2_, 0.2 Mt SO_2_, and 0.4 Mt NO_x_ come from that trade pattern.

Australia produces 86.7 Mt CO_2_, 1.9 Mt SO_2_, and 1.0 Mt NO_x_ in international trade, which, respectively, account for 22%, 39%, and 28% of the total pollutant emissions. Its final product trade caused the least emissions. About 13.1 Mt CO_2_, 0.2 Mt SO_2_, and 0.2 Mt NO_x_ come from the final product trade. Intermediate product trade in the last stage of production produces the most emissions: 49.3 Mt CO_2_, 1.1 Mt SO_2_, and 0.5 Mt NO_x_.

In 2014, the trade-embodied emissions in Japan were 235.7 Mt CO_2_, 1.0 Mt SO_2_, and 1.1 Mt NO_x_. Intermediate product trade caused the most pollutants emissions: about 145 Mt CO_2_, 0.6 Mt SO_2_, and 0.7 Mt NO_x_.

### 4.2. Assessment of the Global Pollution Haven Phenomenon

#### 4.2.1. Changing Trends of Global Pollution Haven under Three Trade Patterns

The pollution haven is reflected by the emissions difference between trade and no-trade scenarios. If the difference is greater than zero, it indicates trade flows (trade liberalization) could increase pollutants emissions, lead to pollution haven. If the difference is less than zero, that means that trade could avoid the country becoming a pollution haven.

**Figure 4:**
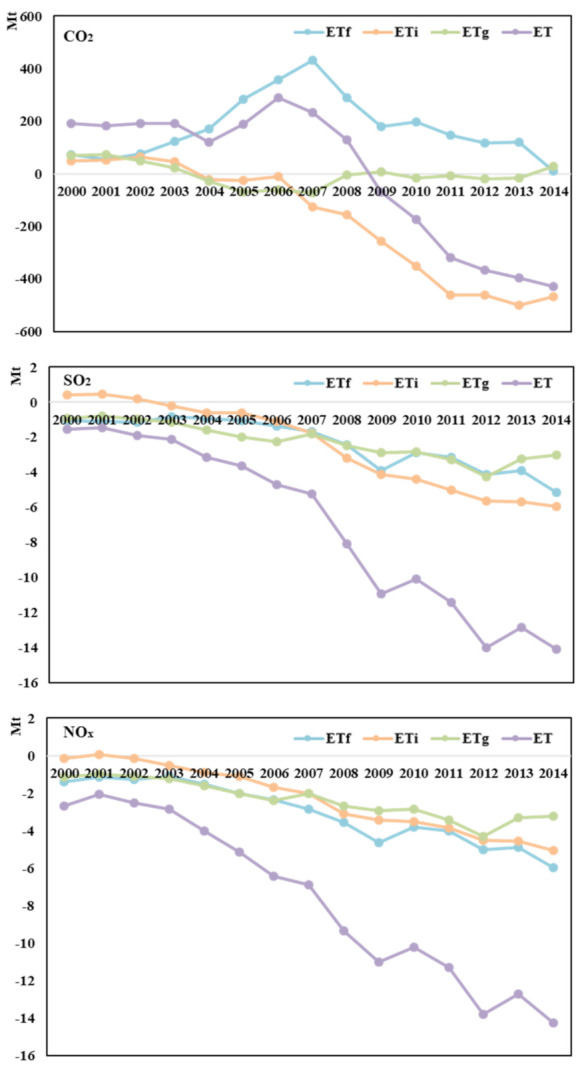
Global pollution haven under different trade patterns from 2000 to 2014. See the details from Appendix E Table A5.

As can be clearly seen from Figure 4, trade could reduce pollutants emissions in general. In 2000, international trade increased 191 million tons of CO_2_ in the world. While in 2014, international trade could reduce 427.7 million tons of CO_2_. For the other two pollutants, trade avoided about 1.5 million tons of SO_2_ and 2.7 million tons of NO_x_ global emissions in 2000. As time goes by, the trade effect in avoiding emissions has become more and more obvious. The avoided emissions of SO_2_ and NO_x_ reached 14.1 million tons and 14.2 million tons in 2014.

The effect of international trade in intermediate product trade on emission reduction is becoming more and more significant. In 2014, global CO_2_ emissions increased 9.8 million tons through final product trade, and 5.2 million tons of SO_2_ and 6.0 million tons of NO_x_ are avoided by the trade pattern. However, intermediate product trade could avoid 437 million tons of CO_2_, 8.9 million tons of SO_2_, and 8.3 million tons of NO_x_ in 2014. This shows that intermediate product trade is more conducive to reducing pollutants emissions. It may because the fragmentation of global production could optimize the international labor division and carry out the reasonable industrial transfer so as to reduce global pollutants emissions.

Final product trade can reduce emissions, but the impact is weak. Final product trade gradually contributes to CO_2_ emissions until 2007. When it peaked in 2007, it increases about 430 million tons of CO_2_ to global emissions. After that, the promoting effect wears off. For SO_2_ and NO_x_, compared with 2000, final product trade reduced 4.1 million tons of SO_2_ and 4.6 million tons of NO_x_ in 2014.

#### 4.2.2. Regional Situations of Pollution Haven

**Figure 5:**
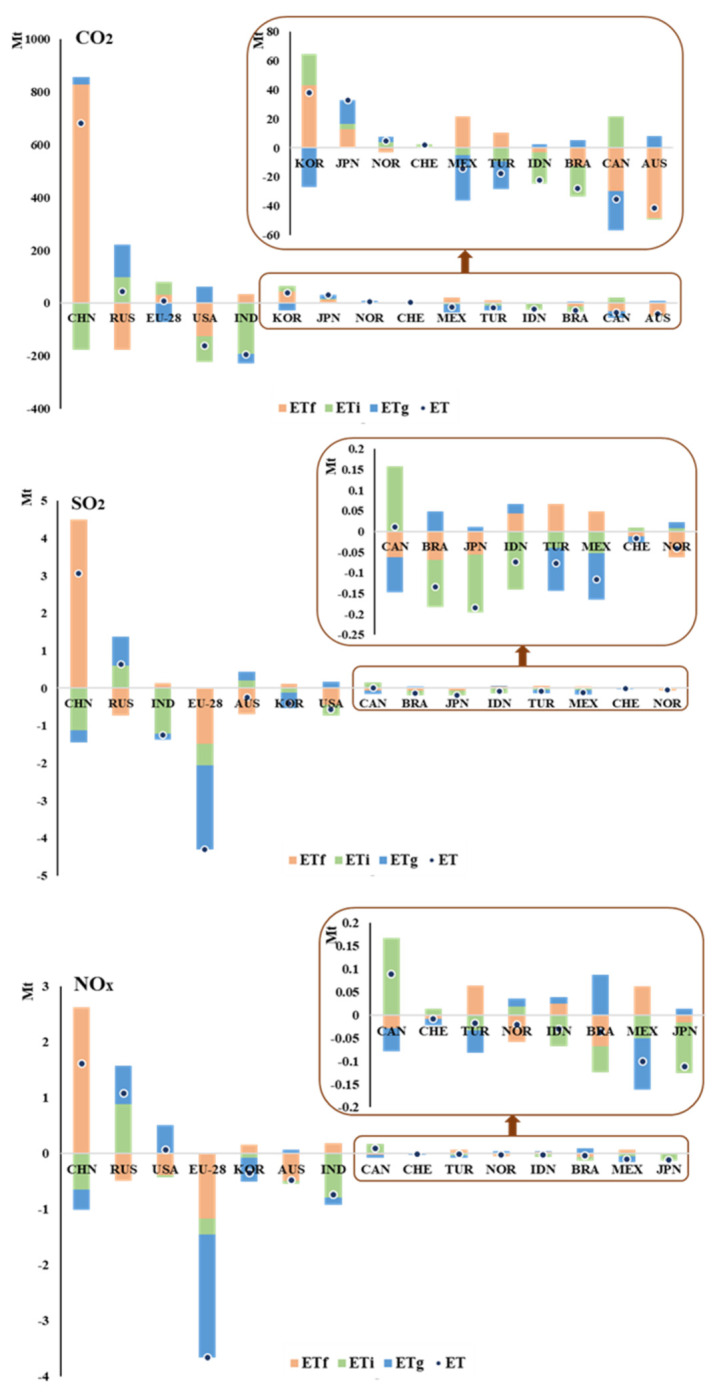
Assess major countries in different patterns of pollution haven. See Table A6 and Table A7 for the full name of the countries.

As can be seen from Figure 5, most countries have avoided domestic pollutants emissions through international trade to varying degrees. The typical countries include the European Union and India. China and Russia are the typical countries that become pollution haven in global trade.

The EU avoids 4.3 million tons of SO_2_ and 3.7 million tons of NO_x_ in global trade in 2014. All three trade patterns could inhibit pollutants emissions, but the trade related to the global value chain’s inhibiting effect is the most obvious. The suppression of SO_2_ and NO_x_ in this pattern accounts for 52% and 60% of the total embodied emissions. As for CO_2_, the EU’s global value chain trade avoids 48.3 million tons of CO_2_. However, on the whole, the EU still increased 8.4 million tons of CO_2_ through trade. Most of the industries in the EU avoid becoming a pollution haven in global trade. Typical industries are agriculture, manufacturing, and mining industries. More than 95% of the suppressed emissions come from these three industries. In addition, the study found the transport industry could contribute significantly to the EU’s NO_x_ emissions. About 85% increased NO_x_ comes from transportation, and it could add 761.3 tons of NO_x_.

As can be seen from Figure 5, India avoids the pollutants emissions mainly through intermediate product trade. India has avoided 230 million tons of CO_2_, 1.4 million tons of SO_2_ and 917 million tons of NO_x_ through that trade pattern in 2014. Its final product trade pattern could increase emissions. This might because India’s domestic infrastructure is currently weak, and most industries are production lines downstream. It still needs to import a large number of technology-intensive goods to meet domestic demand. The mining, electricity, and manufacturing sectors are the main drivers for India avoiding becoming a pollution haven. Almost all the avoided emissions are from these sectors, and about 205.5 million tons of CO_2_, 1.3 million tons of SO_2_ and 789.3 tons of NO_x_ are suppressed through the three industries.

China has increased its most emissions through the final product trade pattern. It becomes the country of worst pollution haven, and most of its industries become pollution haven in global trade. In 2014, China increased about 830 million tons of CO_2_ through final product trade, accounting for 96.7% of the total increased emissions. The increased SO_2_ and NO_x_ emissions are all from the final product trade, which are 4.5 million tons and 2.6 million tons, respectively. China’s manufacturing and electricity industries are the main industries leading to pollution haven, and their emissions are mainly increased through final product trade. This mainly because China is the largest manufacturing country and takes coal as the main energy source, which may produce a large number of pollutants in global trade [56]. The mining industry is almost the only industry in China that avoids emissions. It avoids pollutants mostly through intermediate product trade. China’s mining industry avoids 88.6 million tons of CO_2_, 1 million tons of SO_2_, and 877 tons of NO_x_ in 2014. Overall, China is responsible for more air pollutants than other countries in global trade.

Russia is the country with the second most serious pollution haven. As can be clearly seen from Figure 5, it increases pollutants emissions entirely through intermediate product trade. The increased emissions of CO_2_, SO_2_, and NO_x_ through that trade pattern are 220 million tons, 1.4 million tons, and 1.6 million tons. It might because the trade of the country is related to its natural environment. Russia is rich in forest, oil, and gas resources. There may be pollutants embodied in the extraction of these natural resources [57]. Some raw materials and semi-finished products of energy will be exported in the trade. Most of Russia’s industries become pollution havens in trade. The electricity industry is the most obvious example of a CO_2_ pollution haven. The industry accounts for 60% of the total increased CO_2_ and increases Russia 62.58 million tons of CO_2_ emissions. About 80% of the increased NO_x_ comes from the transportation industry, and it adds about 950 tons of NO_x_.

Overall, Brazil could avoid becoming a pollution haven in global trade. The country avoided 27.7 million tons of CO_2_, 0.13 Mt SO_2_, and 0.04 Mt NO_x_ through trade. Traditional Ricardian trade patterns in Brazil could avoid most emissions. In addition, manufacturing, electricity, transport, and commercial and public services sectors could totally avoid the country’s 32.9 billion tons of CO_2_, 126.7 Mt SO_2_, and 214.6 Mt NO_x_ emissions.

Australia avoids its emissions mainly through final product trade. The trade pattern could avoid the country’s 48.5 Mt CO_2_, 0.7 Mt SO_2_, and 0.5 Mt NO_x_ emissions. It avoids most domestic emissions through the manufacturing industry. The industry decreased 57.6 billion tons of CO_2_, 420 Mt SO_2_, and 720 Mt NO_x_.

In general, the U.S. avoids becoming a pollution haven mainly through final product trade. The avoided emissions under that trade pattern are 125.11 million tons of CO_2_, 450 tons of SO_2_, and 390 tons of NO_x_. The manufacturing and electricity industries in the U.S. avoid becoming a pollution haven. The U.S. manufacturing industry avoids 120.3 million tons of CO_2_, 490.4 tons of SO_2_, and 529.3 tons of NO_x_ through final product trade in 2014. The electricity industry avoids 48.3 million tons of CO_2_, 129.4 tons of SO_2_, and 87.2 tons of NO_x_ through that trade pattern. This might be because the U.S.’s consumption of the final product is large. It is mainly involved in the global value chain and other trade-related activities. Agriculture, transport, commercial service industries could contribute to the emissions in the U.S. The commercial service industry contributes the most to CO_2_ emissions in the U.S. This industry contributes 15.6 million tons of CO_2_ in 2014, mainly through the trade of the global value chain. It might because the U.S. is a big importer and exporter country in service, and its commercial service is developed. The transport industry is the largest contributor to SO_2_ and NO_x_ emissions in the U.S., and it mainly promotes emissions through intermediate product trade. In 2014, intermediate product trade in the transport industry increased 62 tons of SO_2_ and 593 tons of NO_x_, accounting for about 86% of the total promoted emissions in the industry.

Canada’s increased emissions come from intermediate product trade in the last stage of production. This pattern contributes 21.6 million tons of CO_2_, 157 tons of SO_2_, and 168 tons of NO_x_ emissions. It may because that Canada is rich in natural resources and has a small population. The country not only exports forest products and natural resources but also actively participates in the commodities production process.

South Korea mainly reduces three pollutants emissions through the trade related to the global value chain. The trade pattern avoids 26.7 million tons of CO_2_, 412 million tons of SO_2_ and 348 million tons of NO_x_ emissions [58].

## 5. Conclusions

In recent years, the rapid development of trade globalization has promoted the global flow of CO_2_, SO_2_, and NO_x_, which has aroused people’s concerning the trade-related environmental problems such as the pollution haven phenomenon. In this paper, the global pollution haven hypothesis is assessed by investigating the emissions of CO_2_, SO_2_, and NO_x_ in three trade patterns from 2000 to 2014. Based on the environmental-extend MRIO model, the study divides the trade mode into three specific patterns: final product trade, intermediate product trade in the last stage of production, and the trade related to the global value chain.

The pollutants emissions embodied in global trade increased in 2000–2014. Compared with the emissions in 2000, trade increased 2.8 billion tons of CO_2_, 11.4 million tons of SO_2_, and 8.5 million tons of NO_x_ in 2014. Specifically, we found that emissions from intermediate trade are larger, about twice those in final product trade. China, the U.S., the European Union, and India are major pollutants sources in international trade, but their main pollutants drivers are different. China has undertaken international downstream processing and production to meet the final demand of other countries. Its final product trade produces the most pollutants, and the pollutants mainly come from the manufacturing and electricity industries. Intermediate product trade in the U.S. produces the most pollutants. The main emissions of CO_2_ and SO_2_ embodied in the U.S. trade are from the manufacturing industry, while NO_x_ embodied in trade are mainly from the transport industry. India emits pollutants through the traditional Ricardian trade pattern. Emissions from the EU’s three trade patterns are roughly equal.

In general, trade could reduce global CO_2_, SO_2_, and NO_x_ emissions. Global trade could avoid 427.7 million tons of CO_2_, 14.1 million tons of SO_2_, and 14.2 million tons of NO_x_ in 2014. Compared with the final product trade, the emission reduction effect of intermediate product trade becomes more and more significant over time. The European Union avoids its emissions mainly through the trade related to the global value chain, while India avoids becoming a pollution haven through intermediate product trade. Countries such as China and Russia have increased pollutants emissions and become pollution haven in trade. China becomes the worst pollution haven through final product trade. The manufacturing and electricity industries of final product trade are the main pollutants drivers for China. Intermediate product trade in the mining industry could avoid China becoming a pollution haven. Russia becomes the second most polluted haven mainly through intermediate product trade. In addition, it is also found that Canada has become a pollution haven through intermediate product trade in the last stage of production, and South Korea has avoided its emissions by the trade related to the global value chain. Most industries in the U.S. could reduce the emissions in international trade, and its traditional trade pattern in manufacturing and electricity is the main reason why the U.S. could restrain pollutants. The trade related to the global value chain in commercial and public services is the main driver for the U.S. to become a CO_2_ pollution haven. Intermediate product trade in the transport industry leads to the U.S. become a pollution haven of SO_2_ and NO_x_.

There are several potential extensions for this research. First, the MRIO model used in this paper uses a fixed coefficient to represent the production system. This linear representation method actually ignores the price’s inevitable influence in the pursuit of optimization [59]. Therefore, there may be some deviation between the calculated results and the actual situation. In the future, studies are expected to consider the results’ sensitivity according to the price difference. Second, under no-trade scenarios, the study assumed that the original imported products are produced with domestic technology. However, the assumption may overestimate the technological level and natural resources of some importing countries. In the future, more realistic technical assumptions and resource distribution should be established for the accountings under no-trade scenarios. Third, future studies are expected to update the input-output table based on system optimization. It is suggested that studies could apply the method to assess recent pollution havens, such as the trade war between China and the U.S., the trade dispute of regional tariffs and non-tariffs, the control of the U.S.’s export tariffs, and the effects of COVID-19.

## Figures and Tables

**Table 1:** The structure of the multi-regional input-output model.

.		Intermediate Product Trade				Final Demand				Total Outputs
		C^1^	C^2^	…	C^m^	C^1^	C^2^	…	C^m^	
Intermediate Input	C^1^	Z^11^	Z^12^	…	Z^1m^	Y^11^	Y^12^	…	Y^1m^	X^1^
	C^2^	Z^21^	Z^22^	…	Z^2m^	Y^21^	Y^22^	…	Y^2m^	X^2^
	⋮	⋮	⋮	⋮	⋮	⋮	⋮	⋮	⋮	⋮
	C^m^	Z^m1^	Z^m2^	…	Z^mm^	Y^m1^	Y^m2^	…	Y^mm^	X^m^
Value added		V^1^	V^2^	…	V^m^					
Total Inputs		X^1^	X^2^	…	X^m^					

## Data Availability

The data that support the findings of this study could be found in official statistics of World Input-Output Database at http://wiod.org/home and The Eora Global Supply Chain Database at https://worldmrio.com/.

## Appendix A

**Table A1:** Regions in world input-output table.

Region	Abbr.	Region	Abbr.
Canada	CAN	Indonesia	IND
China	CHN	Brazil	BRA
South Korea	KOR	India	IDN
Mexico	MEX	Australia	AUS
Japan	JPN	Turkey	TUR
Russia	RUS	Rest of World	ROW
Switzerland	CHE	Kingdom of Norway	NOR

## Appendix B

**Table A2:** EORA and WIOD departments transform the corresponding table.

EORA	WIOD	
1 Agriculture	1	Crop and animal production, hunting, and related service activities
	2	Forestry and logging
2 Fishing	3	Fishing and aquaculture
3 Mining and Quarrying	4	Mining and quarrying
4 Food and Beverages	5	Manufacture of food products, beverages, and tobacco products
5 Textiles and Wearing Apparel	6	Manufacture of textiles, wearing apparel and leather products
6 Wood and Paper	7	Manufacture of wood and of products of wood and cork, except furniture; manufacture of articles of straw and plaiting materials
	8	Manufacture of paper and paper products
	9	Printing and reproduction of recorded media
7 Petroleum, Chemical, and Non-Metallic Mineral Products	10	Manufacture of coke and refined petroleum products
	11	Manufacture of chemicals and chemical products
	12	Manufacture of basic pharmaceutical products and pharmaceutical preparations
	13	Manufacture of rubber and plastic products
	14	Manufacture of other non-metallic mineral products
8 Metal Products	15	Manufacture of basic metals
	16	Manufacture of fabricated metal products, except machinery and equipment
9 Electrical and Machinery	17	Manufacture of computer, electronic and optical products
	18	Manufacture of electrical equipment
	19	Manufacture of machinery and equipment n.e.c.
10 Transport Equipment	20	Manufacture of motor vehicles, trailers, and semi-trailers
	21	Manufacture of other transport equipment
11 Other Manufacturing	22	Manufacture of furniture; other manufacturing
12 Recycling	23	Repair and installation of machinery and equipment
13 Electricity, Gas and Water	24	Electricity, gas, steam, and air conditioning supply
	25	Water collection, treatment, and supply
23 Education, Health and Other Services	26	Sewerage; waste collection, treatment, and disposal activities; materials recovery; remediation activities and other waste management services
14 Construction	27	Construction
15 Maintenance and Repair	28	Wholesale and retail trade and repair of motor vehicles and motorcycles
16 Wholesale Trade	29	Wholesale trade, except motor vehicles and motorcycles
17 Retail Trade	30	Retail trade, except motor vehicles and motorcycles
19 Transport	31	Land transport and transport via pipelines
	32	Water transport
	33	Air transport
	34	Warehousing and support activities for transportation
20 Post and Telecommunications	35	Postal and courier activities
18 Hotels and Restaurants	36	Accommodation and food service activities
6 Wood and Paper	37	Publishing activities
23 Education, Health and Other Services	38	Motion picture, video, and television program production, sound recording, and music publishing activities; programming and broadcasting activities
20 Post and Telecommunications	39	Telecommunications
21 Financial Intermediation and Business Activities	40	Computer programming, consultancy, and related activities; information service activities
	41	Financial service activities, except insurance and pension funding
	42	Insurance, reinsurance, and pension funding, except compulsory social security
	43	Activities auxiliary to financial services and insurance activities
	44	Real estate activities
	45	Legal and accounting activities; activities of head offices; management consultancy activities
	46	Architectural and engineering activities; technical testing and analysis
	47	Scientific research and development
	48	Advertising and market research
	49	Other professional, scientific, and technical activities; veterinary activities
	50	Administrative and support service activities
22 Public Administration	51	Public administration and defense; compulsory social security
23 Education, Health and Other Services	52	Education
	53	Human health and social work activities
	54	Other service activities
24 Private Households	55	Activities of households as employers; undifferentiated goods- and services-producing activities of households for own use
26 Re-export and Re-import	56	Activities of extraterritorial organizations and bodies
25 Others		It is distributed to the 56 departments on average

## Appendix C

**Table A3:** Time evolution of emissions under the three trade patterns from 2000 to 2014. (Mt).

	CO_2_				SO_2_				NO_x_			
	Tf	Ti	Tg	Trade Percentage *	Tf	Ti	Tg	Trade Percentage *	Tf	Ti	Tg	Trade Percentage *
2000	1932.01	2240.45	1253.25	22.02%	15.86	19.09	10.22	29.51%	15.93	17.88	9.22	28.52%
2001	1989.63	2205.11	1230.15	21.73%	15.78	18.37	9.87	29.48%	15.65	17.08	8.83	28.29%
2002	2056.19	2260.90	1260.30	21.98%	15.83	18.34	9.82	29.71%	15.82	17.20	8.93	28.37%
2003	2242.11	2426.29	1383.97	22.76%	16.31	18.49	10.26	30.05%	15.89	17.40	9.33	28.42%
2004	2490.85	2580.88	1561.62	23.85%	17.60	19.51	11.45	31.36%	16.85	18.22	10.27	29.60%
2005	2697.02	2722.86	1674.74	24.64%	18.13	20.19	12.04	32.07%	17.34	18.58	10.79	30.01%
2006	2922.65	2863.24	1862.63	25.68%	18.75	20.82	13.22	32.96%	17.79	19.03	11.64	30.42%
2007	3050.98	2981.73	1945.31	25.78%	18.63	21.13	13.51	32.52%	18.20	19.90	12.10	29.94%
2008	2930.63	3018.21	1961.95	25.43%	18.51	21.37	13.59	32.01%	17.28	19.59	12.01	30.23%
2009	2655.90	2700.49	1569.23	22.51%	17.22	19.79	11.14	28.62%	16.10	18.17	9.96	27.43%
2010	2930.72	2956.12	1871.91	23.82%	18.60	21.34	13.13	29.97%	17.15	19.39	11.57	28.60%
2011	3016.74	3093.67	2048.44	24.27%	19.16	22.49	14.40	30.46%	17.64	20.43	12.58	29.28%
2012	2988.29	3134.58	2006.94	23.83%	19.00	23.00	14.10	29.77%	17.65	20.91	12.54	28.61%
2013	2935.34	3204.96	2023.07	23.52%	18.74	23.34	14.14	29.27%	17.50	20.99	12.41	28.17%
2014	2941.09	3232.73	2027.31	23.41%	18.85	23.48	14.27	28.94%	17.71	21.29	12.53	27.88%

Trade percentage * means the percentage of trade emissions in total emissions.

## Appendix D

**Table A4:** The situation of global pollutant emissions in 2014. (Mt).

CO_2_	AUS	BRA	CAN	CHE	CHN	IDN	IND	JPN	KOR	MEX	NOR	RUS	TUR	USA	EU-28
Domestic	307.92	488.53	378.22	27.63	8333.12	374.55	1845.48	1035.91	404.59	356.89	25.35	1220.40	245.73	4953.05	2268.99
Tf	13.15	19.08	69.58	5.41	1313.47	33.92	139.12	90.29	78.83	60.86	2.90	55.73	43.14	153.98	416.19
Ti	49.37	31.98	106.23	4.71	876.66	46.40	142.17	87.52	84.69	45.73	8.68	235.58	39.86	163.95	398.21
Tg	24.14	15.50	33.67	2.99	537.57	24.99	76.72	57.92	53.13	15.75	7.36	169.65	25.79	118.15	340.51
SO_2_	AUS	BRA	CAN	CHE	CHN	IDN	IND	JPN	KOR	MEX	NOR	RUS	TUR	USA	EU-28
Domestic	2.88	2.05	1.17	0.14	46.58	2.77	11.39	3.03	0.88	1.63	0.25	3.97	1.37	9.12	9.27
Tf	0.21	0.10	0.25	0.06	7.26	0.26	0.89	0.39	0.24	0.19	0.04	0.23	0.30	0.44	2.73
Ti	1.12	0.22	0.62	0.05	5.40	0.33	0.98	0.41	0.32	0.16	0.07	1.31	0.26	0.49	2.59
Tg	0.55	0.08	0.22	0.03	1.03	0.14	0.18	0.19	0.17	0.06	0.06	1.21	0.10	0.48	1.88
NO_x_	AUS	BRA	CAN	CHE	CHN	IDN	IND	JPN	KOR	MEX	NOR	RUS	TUR	USA	EU-28
Domestic	2.43	3.73	1.20	0.15	27.26	2.63	10.66	3.39	1.16	2.44	0.30	3.89	1.23	14.82	11.20
Tf	0.22	0.19	0.30	0.06	4.29	0.20	0.73	0.41	0.31	0.23	0.05	0.22	0.23	1.04	3.43
Ti	0.51	0.39	0.47	0.05	3.34	0.27	0.75	0.44	0.40	0.16	0.09	1.33	0.17	1.32	3.25
Tg	0.23	0.18	0.15	0.03	1.91	0.13	0.39	0.28	0.22	0.05	0.07	0.85	0.11	0.79	2.55

## Appendix E

**Table A5:** Time evolution of global pollution haven under different trade patterns. (Mt).

	CO_2_				SO_2_				NO_x_			
	ETf	ETi	ETg	ET	ETf	ETi	ETg	ET	ETf	ETi	ETg	ET
2000	74.01	47.98	69.37	191.36	−1.05	0.44	−0.91	−1.53	−1.40	−0.14	−1.15	−2.69
2001	54.28	53.57	74.42	182.27	−1.11	0.45	−0.77	−1.44	−1.13	0.05	−0.99	−2.07
2002	77.16	64.73	49.85	191.74	−1.14	0.21	−0.95	−1.88	−1.27	−0.14	−1.11	−2.52
2003	124.30	45.07	21.85	191.23	−0.81	−0.20	−1.14	−2.14	−1.09	−0.54	−1.24	−2.87
2004	171.44	−23.10	−27.52	120.83	−0.94	−0.60	−1.60	−3.14	−1.51	−0.88	−1.62	−4.01
2005	283.22	−26.50	−68.51	188.21	−1.05	−0.59	−2.00	−3.63	−2.03	−1.09	−2.01	−5.13
2006	359.26	−9.04	−60.97	289.26	−1.36	−1.10	−2.25	−4.71	−2.36	−1.67	−2.38	−6.41
2007	431.47	−126.66	−72.44	232.36	−1.66	−1.78	−1.80	−5.25	−2.86	−2.02	−2.02	−6.89
2008	288.46	−156.44	−3.89	128.13	−2.44	−3.18	−2.48	−8.11	−3.58	−3.08	−2.68	−9.34
2009	179.62	−255.31	6.98	−68.72	−3.88	−4.14	−2.90	−10.92	−4.63	−3.45	−2.92	−11.00
2010	196.48	−352.28	−17.23	−173.04	−2.87	−4.40	−2.83	−10.10	−3.82	−3.53	−2.86	−10.22
2011	147.39	−460.00	−7.49	−320.09	−3.14	−5.01	−3.27	−11.41	−4.02	−3.83	−3.45	−11.30
2012	116.96	−462.28	−20.10	−365.42	−4.12	−5.62	−4.26	−14.00	−5.01	−4.49	−4.31	−13.81
2013	119.31	−501.18	−14.78	−396.66	−3.92	−5.70	−3.22	−12.84	−4.87	−4.54	−3.29	−12.70
2014	9.79	−466.02	28.57	−427.66	−5.15	−5.96	−2.99	−14.10	−5.97	−5.04	−3.23	−14.24

## Appendix F

**Table A6:** Assess major countries in different patterns of pollution haven.

CO_2_	AUS	BRA	CAN	CHE	CHN	IDN	IND	JPN	KOR	MEX	NOR	RUS	TUR	USA	EU−28
ETf	−48.47	−12.56	−29.67	0.63	829.40	−2.89	35.12	12.91	43.32	21.73	−2.89	−176.70	10.43	−125.11	31.92
ETi	−0.77	−20.65	21.61	1.77	−175.99	−21.63	−192.90	3.78	21.37	−4.90	3.79	99.49	−9.33	−97.49	48.28
ETg	7.96	5.55	−27.16	−0.30	28.23	2.56	−36.25	16.07	−26.73	−31.12	3.81	122.66	−18.74	61.53	−71.83
ET	−41.28	−27.67	−35.22	2.10	681.63	−21.96	−194.03	32.76	37.96	−14.29	4.71	45.46	−17.64	−161.06	8.37
SO_2_	AUS	BRA	CAN	CHE	CHN	IDN	IND	JPN	KOR	MEX	NOR	RUS	TUR	USA	EU−28
ETf	−0.69	−0.07	−0.06	−0.01	4.50	0.04	0.13	−0.06	0.12	0.05	−0.06	−0.74	0.07	−0.45	−1.49
ETi	0.21	−0.12	0.16	0.01	−1.12	−0.14	−1.21	−0.14	−0.11	−0.05	0.01	0.60	−0.04	−0.28	−0.57
ETg	0.23	0.05	−0.08	−0.01	−0.32	0.02	−0.17	0.01	−0.41	−0.11	0.01	0.77	−0.10	0.17	−2.23
ET	−0.25	−0.13	0.01	−0.02	3.07	−0.07	−1.25	−0.19	−0.41	−0.12	−0.04	0.64	−0.08	−0.56	−4.30
NO_x_	AUS	BRA	CAN	CHE	CHN	IDN	IND	JPN	KOR	MEX	NOR	RUS	TUR	USA	EU−28
ETf	−0.49	−0.07	−0.03	−0.01	2.62	0.02	0.18	−0.02	0.16	0.06	−0.06	−0.49	0.06	−0.39	−1.17
ETi	−0.05	−0.06	0.17	0.01	−0.65	−0.07	−0.80	−0.11	−0.08	−0.05	0.02	0.88	−0.03	−0.04	−0.28
ETg	0.06	0.09	−0.05	−0.01	−0.37	0.01	−0.12	0.01	−0.43	−0.11	0.02	0.69	−0.05	0.50	−2.22
ET	−0.48	−0.04	0.09	−0.01	1.61	−0.03	−0.73	−0.11	−0.35	−0.10	−0.02	1.07	−0.02	0.07	−3.66

## Appendix G

**Table A7:** Pollution haven phenomena in different sectors.

CO_2_	AUS	BRA	CAN	CHE	CHN	IDN	IND	JPN	KOR	MEX	NOR	RUS	TUR	USA	EU−28	ROW
Agriculture	813.19	3976.70	1854.38	−137.59	2996.18	936.06	4033.69	−2732.31	−1597.31	−34.36	61.47	−2083.28	567.14	491.56	−613.41	8425.01
Manufacturing	−57,588.04	−23,073.34	−63,620.99	407.94	53,2977.02	−21,007.52	−36,690.88	29,970.51	41,533.65	−8203.06	−2849.19	−56,165.31	−15,664.37	−12,0300.09	1544.10	−37,9453.55
Electricity, Gas and Water Supply	2074.25	−3293.04	4448.36	308.63	18,5483.83	−5441.27	−62,120.30	16,019.77	15,590.92	−4148.18	87.90	62,580.06	−727.73	−48,270.66	15,641.47	−97,308.84
Construction	20.92	28.51	1305.56	110.94	3887.99	16.53	−1561.24	107.26	−11.31	1.04	−16.76	−1516.99	501.09	−2293.87	6820.89	−10,237.38
Transport	244.55	−139.32	1250.06	111.73	5497.87	−45.96	40.15	671.33	194.85	−127.75	396.55	4982.04	200.16	752.43	4511.68	−33,758.87
Commercial and public services	1591.08	−6364.98	3383.50	1457.45	39,413.87	−1002.58	8927.49	5730.73	3744.05	−5033.00	−376.61	9142.89	−612.86	15,597.07	34,253.55	−31,4592.18
Mining	11,568.51	1197.34	16,162.95	−159.57	−88,622.29	4580.67	−10,6654.60	−17,011.83	−21,493.63	3258.08	7407.98	28,517.10	−1901.02	−7041.07	−53,791.49	99,421.61
**SO2**	**AUS**	**BRA**	**CAN**	**CHE**	**CHN**	**IDN**	**IND**	**JPN**	**KOR**	**MEX**	**NOR**	**RUS**	**TUR**	**USA**	**EU−28**	**ROW**
Agriculture	35.7952	21.3613	7.7993	−1.4989	−19.1006	5.2070	13.6751	−40.7338	−5.1687	0.2891	0.3317	−4.9737	3.9346	0.4968	−115.3375	225.1355
Manufacturing	−419.6564	−128.8580	−81.4062	−21.1871	2265.4992	−50.1084	−441.2877	86.8428	143.9538	−69.7924	−75.4548	240.9774	−66.8601	−490.4393	−2234.0749	−6703.6796
Electricity, Gas and Water Supply	6.4346	−20.7446	26.0914	1.7993	1303.8631	−43.4605	−419.5561	20.7369	21.5036	−30.4084	1.1004	132.3467	−5.2215	−129.3633	−36.5816	−759.1605
Construction	0.1249	0.0306	0.2534	0.0509	8.6222	0.0770	−2.3360	0.1869	−0.0043	0.0012	−0.0867	−0.4584	0.5884	−0.6918	6.4777	−32.5642
Transport	14.6954	−8.4921	41.3893	3.8677	489.9809	−6.3706	1.2387	30.2807	14.0549	−7.4099	16.4234	225.9102	7.5311	72.7504	202.0840	−1826.5121
Commercial and public services	5.8808	−4.5674	4.7393	8.2517	48.7309	−1.3043	16.2994	6.2934	3.4564	−12.7214	−1.8814	9.7761	−1.7460	4.3198	128.0736	−1715.8128
Mining	103.5567	6.6260	12.0093	−7.4649	−1032.0021	21.6392	−413.5721	−288.7251	−584.9262	3.7101	19.2519	37.0912	−14.5321	−16.8695	−2249.7721	403.2461
**NOx**	**AUS**	**BRA**	**CAN**	**CHE**	**CHN**	**IDN**	**IND**	**JPN**	**KOR**	**MEX**	**NOR**	**RUS**	**TUR**	**USA**	**EU−28**	**ROW**
Agriculture	78.8492	171.5942	12.9246	−2.1648	−7.4253	21.3170	41.5665	−37.4412	−7.7252	−0.4602	−0.5737	−5.4903	8.6264	7.8490	−120.5805	606.1879
Manufacturing	−721.9550	−121.0889	−137.8095	−22.0380	991.8050	−24.8232	−155.1996	56.9885	83.6468	−35.0559	−73.4768	−111.0286	−34.5658	−529.2675	−2188.7304	−4963.7658
Electricity, Gas and Water Supply	6.5073	−14.3876	12.9884	1.5173	543.3247	−23.8803	−291.4806	24.0456	22.1546	−10.3253	1.4330	159.1564	−1.8053	−87.1642	−0.5696	−404.0023
Construction	0.1001	0.0268	0.4671	0.0455	3.4901	0.0367	−1.5451	0.1517	−0.0032	0.0009	−0.0679	−0.4531	0.2502	−0.7650	6.7328	−27.3855
Transport	46.6570	−72.1490	184.9426	13.6321	927.2810	−15.2218	3.8621	79.9189	89.3039	−47.1239	34.4223	952.4031	23.2680	690.7139	761.3043	−5474.3492
Commercial and public services	5.6167	−6.9412	3.2432	7.3121	25.0546	−1.1528	10.4650	3.6508	2.7871	−11.5791	−1.9402	4.3173	−1.3570	6.9247	126.7020	−1647.1545
Mining	101.7744	6.4879	12.5387	−7.1643	−876.9951	13.8994	−342.5723	−239.1472	−538.4078	4.3002	18.6975	73.8852	−12.7312	−19.5288	−2248.7146	388.7168
